# Analyses of Skin Secretions of *Vipera ammodytes* (Linnaeus, 1758) (Reptilia: Serpentes), with Focus on the Complex Compounds and Their Possible Role in the Chemical Communication

**DOI:** 10.3390/molecules25163622

**Published:** 2020-08-09

**Authors:** Kostadin Andonov, Angel Dyugmedzhiev, Simeon Lukanov, Miroslav Slavchev, Emiliya Vacheva, Nikola Stanchev, Georgi Popgeorgiev, Deyan Duhalov, Yurii V. Kornilev, Daniela Nedeltcheva-Antonova, Borislav Naumov

**Affiliations:** 1 Institute of Biodiversity and Ecosystem Research-BAS, 2 Gagarin Street, 1113 Sofia, Bulgaria; angel_diugmedjiev@abv.bg (A.D.); simeon_lukanov@abv.bg (S.L.); slmiro@abv.bg (M.S.); herpetology_bg@yahoo.com (B.N.); 2 National Museum of Natural History-BAS, 1 Tsar Osvoboditel Blvd., 1000 Sofia, Bulgaria; emilia.vacheva@gmail.com (E.V.); georgi.popgeorgiev@gmail.com (G.P.); yukornilev@gmail.com (Y.V.K.); 3 Department of Zoology and Anthropology, Sofia University “St. Kliment Ohridski”, 15 Tsar Osvoboditel Blvd., 1504 Sofia, Bulgaria; nickolastanchev@abv.bg; 4 Bulgarian Society for the Protection of Birds/BirdLife Bulgaria, Yavorov Complex, Bl. 71, Vh. 4, PO Box 50, 1111 Sofia, Bulgaria; 5 Independent researcher, Vrabnica 1 Complex, Bl. 538, Vh. V, ap. 47, 1229 Sofia, Bulgaria; deyan_duchalov@abv.bg; 6 Integrative Zoology, Department of Evolutionary Biology, Faculty of Life Sciences, University of Vienna, Althanstrasse 14, 1090 Vienna, Austria; 7 Institute of Organic Chemistry with Centre of Phytochemistry-BAS, 9 Acad. G. Bonchev Street, 1113 Sofia, Bulgaria; dantonova@orgchm.bas.bg

**Keywords:** pheromone, snakes, behavior, ketones, lipids

## Abstract

Snakes rely heavily on chemical cues when foraging, searching for mates, etc. Snakes’ sex attractiveness pheromones comprise mainly heavy, semi-volatile compounds such as ketones. Here we investigated the composition of skin secretions of adult *Vipera ammodytes* (Linnaeus, 1758) individuals. The samples were analyzed by gas chromatography/mass spectrometry and the identification of the compounds was performed using commercial mass spectral libraries and retention times. The relative concentrations of all detected compounds were tested for significant differences between (1) male vs. female live individuals, (2) shed skin vs. live individuals, and (3) pre-reproductive vs. reproductive live individuals. We detected fifty-nine compounds of which six were ketones. Two ketones (2-pentacosanone and 2-heptacosanone) were present in many of the samples and thus may have an important role in the *V. ammodytes* chemical communication. We did not find significant differences between the relative concentrations of the compounds between male and female individuals (only three compounds are exceptions). Significant differences were found between extracts from shed skins and live individuals and between live pre-reproductive individuals and live reproductive individuals. The results of the study suggest that chemical communication in *V. ammodytes* involves less compounds in comparison to the known literature data for other species.

## 1. Introduction

As a result of past evolutionary adaptations for subterranean life, snakes are generally constrained in the visual perception of their environment [1] and rely heavily on chemical cues when foraging, escaping from predators, and searching for mates [2,3,4]. The vomeronasal system is one of the most developed sensory systems in snakes, in addition to their nasal olfactory system and taste buds [5,6,7]. In contrast to most animals that use chemical signalization produced by a discrete gland, snake pheromones are components of their integumental skin lipids [2,8]. Moreover, the production of most known pheromones in vertebrates, including snakes, is usually controlled by hormones to some extent [9,10,11]. Low temperatures and hibernation may also affect the production of pheromones in snakes [12,13]. This, coupled with their specific behaviors such as secretive lifestyle, complicates the extraction of skin chemicals and pheromones, and has resulted in a few studies on the variety of skin compounds across different species. However, the extensive ecological plasticity of snakes, allowing them to inhabit almost all terrestrial and aquatic habitats, as well as their diverse reproductive strategies and social behaviors, strongly suggests variations in their chemical communication and pheromone composition.

Although interest in snakes’ chemical cues and their respective behaviors has increased in recent decades [4], many groups remain understudied and dearth of information about the chemical ecology and reproductive behavior of many species and still research on chemical signals in animals has centered mainly on insects [14]. There are, however, many studies on other reptiles such as lizards (e.g., on the skin lipids of *Sceloporus virgatus* Smith, 1938 [15], on the role of scent marks in *Podarcis hispanicus* (Steindachner, 1870) [16], on the relation between environmental conditions and chemical signalization in lizards [17] etc.). However, these currently available data are based mostly on a couple of studies, which have only focused on a limited amount of snake species. Additionally, the degree to which pheromonal design is species-specific is heavily understudied. Most studies on snakes’ chemical communications have concentrated on the genus *Thamnophis* [2,4,18,19,20,21,22,23,24]. Additional publications include studies on skin lipids and pheromones of *Drymarchon corais* (Boie, 1827) [25], *Boiga irregularis* (Bechstein, 1802) [26], and species of the genus *Crotalus* [27,28]. The pheromone compounds in the fraction of skin lipids were described in some *Thamnophis* species as a complex of heavy, nonvolatile long-chained saturated and monounsaturated methyl ketones (C29–C37) [2,18,29]. Thus, they can be extracted by body immersion or shed skin soaking in nonpolar solvents such as hexane, benzene, toluene [30]. Additionally, studies suggested the presence of volatile cues in the chemical communication of *Nerodia sipedon* (Linnaeus, 1758) [31] and *Thamnophis sirtalis parietalis* (Linnaeus, 1758) [32], although the vomeronasal system is responsive mainly to nonvolatile chemicals such as lipids, commonly referred to as vomodor [7,33,34,35].

Previous studies have investigated the mating behavior related to chemical communication for both female–male attraction and male-to-male combats in different *Vipera* species [36,37,38], sexual dimorphism of the tongue morphology of *Agkistrodon contortrix* (Linnaeus, 1766) [39], aggregation behavior of *Storeria dekayi* (Holbrook, 1839) with focus on skin secretion [40]. However, there are limited studies focused on the pheromone composition among Old World species, and the Viperidae family including the genus *Vipera*. To extend our knowledge on pheromone communication, we chose to study the chemical composition of the skin secretions of *Vipera ammodytes* (Linnaeus, 1758), focusing on the complex chemical compounds and their variety.

The relatively extensive range of *V. ammodytes*—from the westernmost foothills of the Alps throughout the whole Balkan Peninsula and many Aegean islands, northwestern and northern Asia Minor to the Lesser Caucasus to the east [41]—its specific mating behavior and male-to-male combats [42] and the lack of data for its chemical communication, make the species an interesting target for skin lipids research. We expected differences between individuals based on sex and reproductive status. We also tested whether differences were detectable between shed skins and live specimen samples as a means of evaluating the method of pheromone extraction. We hypothesized that there might be a difference in the two methods, considering the different duration of soaking and the difference in the chemical stability of the potential compounds. Thus, the different methods might reveal different insights into the ecological and behavioral characteristics of the animal.

## 2. Results

**Figure 1:**
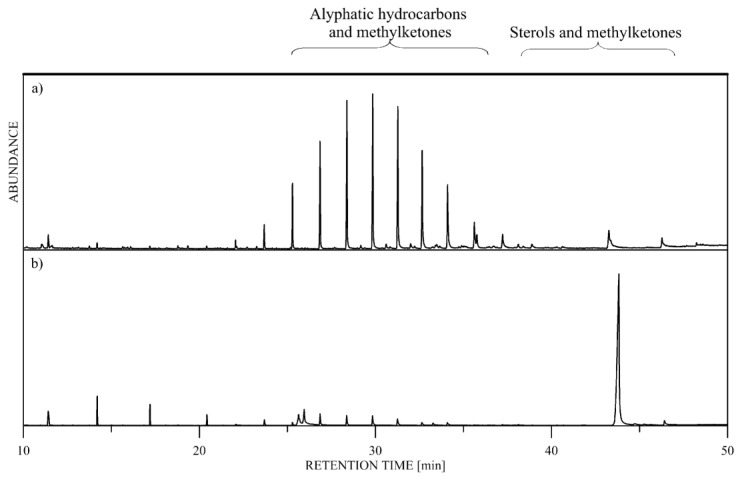
Representative total ion current chromatograms. (a) Reproductive male, live individual sample; (b) male, shed skin sample.

The chemical analyses of the samples provided chromatograms with a complex of skin secretion compounds (*n* = 59; Supplementary Materials 1 and 2). An example of two different chromatograms is presented in Figure 1. The long-chain hydrocarbons (21 compounds, ~36%) and lipids and their derivatives (11 compounds, ~19%) were the most pronounced compounds. Ketones and other aroma compounds, such as aldehydes and phenols, were also detected but in smaller proportions. The ketones we found in *V. ammodytes* are 5,9-undecadien-2-one, 6,10-dimethyl- (C_13_H_22_O), 2-pentadecanone,6,10,14-trimethyl- (C_18_H_36_O), 2-pentacosanone (C_25_H_50_O), 2-heptacosanone (C_27_H_54_O), 2-hentriacontanone (C_31_H_62_O) and 2-tritriacontanone (C_33_H_66_O). The GC/MS analysis revealed that 2-hentriacontanone, cholesterol and a tocopherol isomer have similar chromatographic behavior, therefore, are only partially separated and are observed as overlapping peaks in the chromatograms. This fact makes their quantification difficult (only an approximation of their content in the samples could be made by using characteristic *m*/*z* values) and, therefore, they were not included in the statistical analyses.

Statistically significant differences in the relative concentrations of compounds between live male and female individuals were detected only for *n*-dodecane, *n*-hexadecane, and *n*-octadecane (Table 1, Table 2 and Table 3).

The samples from live individuals and shed skins revealed significant differences in the relative concentrations for some compounds (Table 1, Table 2 and Table 3). The main differences in the concentrations were among the long-chain hydrocarbons and lipid compounds. The only ketone that was significantly different was 2-pentacosanone (*t*-value = −2.2396, *p* = 0.0324); it was the most often detected ketone (in 13 samples, ~36%).

Statistically significant differences between samples from pre-reproductive and reproductive individuals were found in seven alkane compounds (*n*-dodecane, *n*-pentadecane, cyclododecane, *n*-hexadecane, *n*-octadecane, *n*-nonadecane, eicosane) and one aroma compound (phenol, di-tert-butyl-; Table 1, Table 2 and Table 3).

The principal component analysis (PCA) that we performed, showed that the first three components bring more than 50% of the variation (see Supplementary Material 4). The strongest correlations with the three main components, and therefore greater weight for the variance within the samples from live individuals and shed skins and samples from pre-reproductive and reproductive individuals, are found for *n*-dodecane, *n*-pentadecane, *n*-hexadecane, *n*-octadecane, *n*-nonadecane, eicosane, 2-pentacosanone, nonacosane, triacontane, squalene, hentriacontane, cholesterol benzoate, and cholesta-3,5-diene. The strongest correlations with the three main components within the samples from live individuals and skins are found for *n*-dodecane, *n*-tetradecane, *n*-hexadecane, *n*-octadecane, docosane, pentacosane, heptacosane, octacosane, 2-pentacosanone, nonacosane, triacontane, squalene, hentriacontane, and cholesta-3,5-diene.

A positive correlation was detected between the snout–vent length (SVL) and the number of detected compounds in the samples of live individuals (R = 0.545, *p* = 0.003). No significant correlation was found between the body mass and the number of detected compounds in the samples of live individuals (R = 0.286, *p* = 0.148).

## 3. Discussion

The studied samples, according to the GC/MS analysis, represent a complex mixture of compounds, which diversify significantly in chemical structure, respectively, in polarity and volatility. Therefore, the chromatographic methodology we used was carefully selected so as to be particularly suitable for complex mixture analysis. Special attention was paid to the design of the temperature gradient resulting in effective separation of the analytes with very few co-eluting components.

We found various compounds in the skin secretions of *V. ammodytes*. Some of them might have a role in the water permeability in snake molts [43], or even be contaminants from the environment [28]. However, according to literature data, the compounds that are most likely to be involved in the chemical communication in snakes are the ketones [2]. We detected only six ketones overall. In one sample of a male, 2-tritriacontanone was present. The 5,9-undecadien-2-one, 6,10-dimethyl-, and 2-pentadecanone,6,10,14-trimethyl- were both present in two samples of a male and a female, but considering the fact that they are terpenoids, they are most likely artefacts from the environment. In contrast, 2-pentacosanone and 2-heptacosanone were present in many samples from both sexes—thirteen and five, respectively. We speculate that 2-pentacosanone, 2-heptacosanone might play a key role in the chemical communication of *V. ammodytes*. Although we are aware that the GC/MS method might not be able to detect compounds with very high molecular mass that are even less volatile, we speculate that the complex of ketones in the *V. ammodytes* skin secretion is less diverse compared to well-studied species such as *T. sirtalis* [2]. For example, the chemical cues found in female *Thamnophis sirtalis parietalis* are 17 long-chain saturated and unsaturated methyl ketones (C29–C37) representing the sex pheromone, and squalene and other skin extract components in the male recognition pheromone [2,29]. Series of long-chained methyl ketones and ketodienes were also found in *B. irregularis* (C31–C37). Well-pronounced peaks of squalene were also observed in many of the analyzed *V. ammodytes* samples. However, behavior analyses are required in order to determine the compounds that comprise the sex pheromone. We found other aroma compounds such as aldehydes and fatty acid derivatives, which are used as part of the pheromone complex in many invertebrates, such as moths [44,45], and also vertebrates such as Spotted hyenas *Crocuta crocuta* (Erxleben, 1777) [46]. This provides a base for further behavioral research, required to understand which are the compounds that attract other conspecifics. Although the common understanding is that snakes’ sex attractiveness pheromones comprise mainly heavy, semi volatile compounds [2,25,26,27,28], in species such as *N. fasciata* and *T. s. parietalis* airborne components were described as well [31,32]. Thus, we need further testing using alternative methods for chemical analyzes to reveal the whole spectrum of components in the *V. ammodytes* skin lipid secretions.

The free fatty acids, cholesterol, steryl esters and other sterol lipids, which we detected are considered usual for most of the studied species: *Vipera berus* (Linnaeus, 1758), *Echis carinatus* (Schneider, 1801) and *Gloydius halys* (Pallas, 1776) [47], and *Crotalus adamanteus* Palisot de Beauvois, 1799, *C. atrox* Baird and Girard, 1853, *C. horridus* Linnaeus, 1758, *C. vegrandis* Klauber, 1941 and *C. viridis* Rafinesque, 1818 [27], *Thamnophis* spp. [18].

We detected traces of phthalate contamination in few samples, but these compounds are easy to be identified and do not affect the results. These phthalates could be an artifact from the environment or from the gloves we use for handling venomous snakes.

The focus of this stage of the study was exploratory, aiming to describe the general pheromonal characteristics of the species. The intraspecific variation in pheromonal design, widely recognized in vertebrates and also some squamates [48,49], planned as the next research stage, will then be based on a solid foundation.

### 3.1. Comparison of Skin Chemical Compounds between Males and Females

We found many of the compounds considered in other species to be part of the pheromone activity, such as long-chain ketones and squalene, but they were present in both sexes and our results failed to identify significant differences between sexes except for three long-chained alkanes, which are most likely environmental artifacts (see Section 2), and will be a subject for further detailed research. Most above-mentioned studies found differences between male and female secretions in some components. Male individuals of *T. sirtalis* may mimic females in their chemical exposure (called “she-males” by Mason and Crews [50]), where male-oriented courtship is observed [50,51,52,53,54]. However, this behavior is the exception. In addition, we have to consider the fact that the mating behaviors of *T. sirtalis* and *V. ammodytes* are very different. *Thamnophis sirtalis* exhibits the so-called mating balls type of behavior where numerous males are trying to copulate with a single female [55], while in *V. ammodytes* the male-to-male combat is typical [42]. That might be explained with frequent female mimicry of male individuals of *V. ammodytes* or it might be that male-to-male combats are the result of intense male chemical signalization which could be to some extent connected to testosterone as, for example, is registered in *Podarcis muralis* (Laurenti, 1768) [56]. Further research is required on the nature of the skin lipids in males and females of *V. ammodytes* and tests on the correlation of the mating behavior with the chemical communication.

Another reason for the lack of significant difference between male and female skin chemicals composition might be connected to the copulatory plug commonly connected to inhibitory pheromones, making the female less attractive post-copulation [57,58,59]. This might explain why the females we analyzed had lower concentrations of ketones than expected. For further studies, animals in captivity should be used and skin lipid samples should be collected before and after copulation. That would give a strong insight to this rather curious situation.

### 3.2. Comparison of Skin Chemical Compounds between Live Individuals and Skin Sheds

The two sampling methodologies both returned valuable information but seem to be geared to produce different results. Shed skin is useful for quantitative analyses and gives higher concentrations of lipids, since they are inert and stable, and can stay long on the surface of the skin [60]. Thus, these compounds are of high abundance regardless of the season. Therefore, to detect seasonal variation of the skin surface compounds, live individuals must be used. However, we found some significant differences in the relative concentrations as well. In the shed skins, the sterol lipids and their derivatives were the major component, while in the live individual samples the long-chain hydrocarbons were prevailing. The only ketone with significantly different relative concentrations was 2-pentacosanone. Although the relative concentrations of the compounds were generally higher in the shed skin samples, this ketone was found with higher relative concentrations in the live individual samples when present. Given that most shed skin were collected in the mid-summer, long after the mating season, this ketone might have been inhibited somehow, e.g., by a hormone or the previously described copulatory plug and therefore was missing in the sheds. Even if this compound is playing a dominant role in the snakes’ communication, it would be difficult to demonstrate this without checking for both positive and negative correlations with other compounds found in the skin secretions. Considering the fact that we describe only relative concentrations, the difference might occur due to the extraction of very concentrated fatty acids, lipids and others, and therefore reduce the relative concentration of the above-mentioned ketone.

### 3.3. Comparison of Skin Chemical Compounds between Pre-Reproductive and Reproductive Phases

LeMaster and Mason [23] demonstrated annual and seasonal variation in the female attractiveness pheromones. In addition, Parker and Mason [12] related the concentration and composition of pheromones with low temperature dormancy. Uhrig et al., [13] supported the hypothesis of seasonal variation and ecological factors impacting the quality and quantity of pheromones. Moreover, they suggested that the period of exposure to higher temperatures and dry conditions might influence the production of pheromones as a key stimulus in addition to hormonal activity. Thus, our initial hypothesis was that there will be significant differences in the ketones detected in the samples from these two phases. We found significant differences only in hydrocarbons and phenol, di-tert-butyl-. The presence of alkanes in the samples of skin secretions of snakes is recorded in the literature. In the study of Ahern and Dowling [25], they are described as contaminants, representing only 5% of the substances found in the complex. In Baedke et al. [30], the skin lipids are described as highly miscible in nonpolar solvents, and therefore it is easy to find various hydrocarbons among them originating from different sources. One explanation might be that snakes that emerge from hibernation earlier are active for longer periods and have more time to absorb in their integumental lipid layer different compounds from the environment, such as the above-mentioned hydrocarbons. However, this somewhat contradicts the result that there were significantly more hydrocarbons in the live individual samples than from the shed skins samples. We could only speculate that if these hydrocarbons are contaminants from the environment, they can easily detach once the skin is shed.

### 3.4. Correlation between the Number of Detected Compound and SVL

The correlation we found between the number of detected compounds and the SVL is difficult to interpret. Shine et al. [61] established a correlation between the body length and condition and the length of the dominant methyl ketone in the skin secretions in *T. sirtalis*, with concentration of the longest ketone directly proportional to the length of the female snake. Considering that we describe mainly two frequently detected ketones, the described statement by Shine et al. [61] is unlikely to apply in our case, and the level of attractiveness of the individuals could be determined by a combination of various compounds. However, that would be a speculation without detailed behavioral analyses, so this correlation requires further investigation.

## 4. Materials and Methods

### 4.1. Field Work

**Figure 2:**
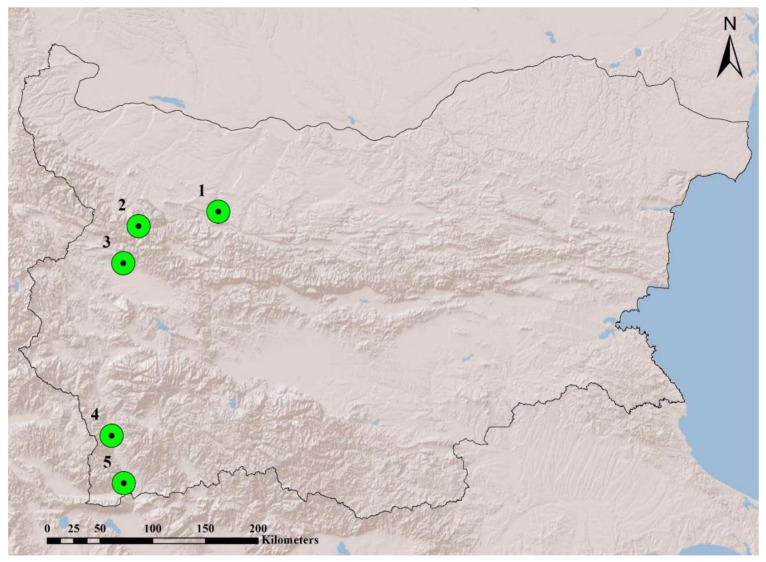
Map with the localities where vipers (V. ammodytes) were collected: 1. Karlukovo Village, 2. Lakatnik Village, 3. Balsha Village, 4. Kresna Gorge, 5. Rupite area.

We investigated only adult individuals of *V. ammodytes* (17 females and 20 males) using both shed skin (*n* = 10) and live snakes (*n* = 27). The data were collected in 2019 from five localities in western Bulgaria—Karlukovo Village, Lakatnik Village, Balsha Village, Kresna Gorge and Rupite area (Figure 2). Samples were obtained from the beginning of March to the first half of June, during the presumed active reproductive season. Snakes were located by active visual search, including inspection of potential hideouts. Immediately after capture, chemical extraction was carried out (see Section 4.2 below). Standard morphometric measurements of each individual were obtained using a measuring tape (precision: 0.5 cm); snakes were weighted using a digital scale (precision: 0.01 g). Sex of individuals was determined by the color pattern of the body, the length and the width of the tail root, as well as the ratio between the snout–vent length and tail length [62]. Individuals were considered as adults when the total body length (Ltot) > 46 cm in females and Ltot > 49 cm in males [63]. Most snakes were released immediately at the site of capture after the extraction procedure was carried out. Individuals were marked with a non-toxic, alcohol-free red color pen (Faber-Castell Multimark 1525 permanent) to avoid repeated sampling during the same visit. Vipers were photo-documented for long-term identification [64].

Additionally, we collected samples from shed skin (see Section 4.2 below). Three freshly shed skins, all from male vipers, were found in the field on 24.04., 25.04. and 05.05. Two male vipers with shedding skin were kept for one day, until shedding was complete and then released at the site of capture. Five individuals were brought to the laboratory where they were kept for 1–1.5 months until they shed their skin, after which they were released back at the site of capture. Each viper was kept in an individual escape-proof, but well-ventilated, plastic box (50 L × 30 W × 30 H cm) with beech chips for substrate. A constantly running, heating coil was provided at one end of the box and a shelter at the other. Temperature was maintained between 30–32 °C during the day (09:00 h to 20:00 h) and maintained at approximately 17 °C during nighttime. Each viper was fed every 10–15 days with a dead laboratory mouse. Water was provided ad libitum.

The samples from live individuals were grouped as coming from pre-reproductive or reproductively active adults. In vipers with spring spermiogenesis (such as *V. ammodytes*), the start of the mating period is characterized by the synchronized shedding of adult males [38,65,66,67]. Thus, individuals sampled before the first shedding of the males (*n* = 7) were attributed to the pre-reproductive group, and those which were sampled after this period (*n* = 20) to the reproductive group.

Research was carried out in accordance with permit NO. 767/24.01.2019 provided by the Ministry of Environment and Waters. No animals were harmed or exhibited signs of deteriorating health, were euthanized, or died in captivity during the research.

### 4.2. Extraction of Lipid Fraction

Skin lipids were extracted both from living specimens and shed skins. Live individuals’ full bodies (excluding the tail and the head) were immersed for 1 min in 300 mL *n*-hexane (C_6_H_14_) [20]. The samples were collected in clean dark glass containers to avoid any contamination from plastic. All caps were packed with parafilm. The snakes were then washed with warm water and dried with a towel. Given the specifics of the field work, some samples of live individuals were stored in the fridge after a few days.

Shed skins were soaked in *n*-hexane for 24 h, in clean dark glass containers with glass cap, following the methodology for extraction in Baedke et al. [30]. Shed skin samples were immediately placed for storage in the fridge.

All samples were concentrated approximately 1:150 times by evaporation of the solvent using a rotary vacuum evaporator to the final sample volume of 2 mL and kept in dark glass vials with screw aluminum caps with 8 mm septum in a fridge (at 5 °C) prior to the analysis.

All glass containers are made of borosilicate glass and the volumetric glassware used is class A, produced according the international standards ASTM 542 and ISO 4787.

### 4.3. Gas Chromatography/Mass Spectrometry (GC/MS)

The GC/MS analysis was carried out on an Agilent 7820A GC System Plus gas chromatograph coupled with 5977B Mass Selective detector and flame ionization detector (Agilent Technologies, Palo Alto, CA, USA) by using a mid-polar capillary column DB-17HT (J&W Scientific, Folsom, CA, USA) with 60 m column length, 0.25 mm i.d., and 0.25 µm film thickness. The oven temperature was programmed from 75 °C (5 min held) to 150 °C at a rate of 10 °C/min, from 150 to 250 °C at a rate of 5 °C/min, from 250 to 275 °C at a rate of 2.5 °C and from 275 to 325 °C at 10 °C/min with application of a 30 min isotherm at the final temperature. As a carrier gas, helium (99.999%) was used at a constant flow rate of 0.8 mL/min. A pulsed splitless mode was used, with injection pulse pressure 25 psi until 0.5 min, the inlet and transfer line temperature were set to 300 °C. Electron ionization (EI) mode at 70 eV electron energy was applied, the ion source temperature was set to 230 °C, and the quadrupole temperature was 150 °C. The mass selective detector operated in scan range 45–1000 *m*/*z*.

Instrument control and data collection were carried out using MassHunter Workstation Software (Revision B.06.07, Agilent Technologies, Santa Clara, CA, USA).

We ran control samples with the same *n*-hexane used for the extraction of the skin lipids, with identical sample preparation protocol as the unknown samples with each samples’ series.

### 4.4. Identification and Quantitative Analysis

The identification of the compounds was performed using commercial mass spectral libraries (NIST 14, Wiley 7th Mass Spectra Register) and retention times (linear retention indices, LRI) as previously described in Nedeltcheva-Antonova et al. [68]. If the corresponding reference data were missing, the structures were suggested using their general fragmentation pattern and reference literature mass spectra. The main compound’s quantification was carried out by an internal normalization method (relating the peak area of any compound of interest to the total peak area), with response factor 1 set for all of the sample constituents as described in Nedeltcheva-Antonova et al. [69].

### 4.5. Statistical Analyses

All statistical analyses were performed using software PAST v. 3.25 [70]. We used the relative concentrations of all detected compounds in the solution as dependent variables. To test for significant differences among the designated groups (grouping variables), i.e., (1) male live individuals vs. female, (2) shed skin vs. live individuals (both sexes), and (3) live pre-reproductive individuals vs. live reproductive individuals (both sexes), the chemical concentrations were first tested separately for normality. Since the null hypothesis was rejected, to normalize distribution and to correct the problem of non-independence of proportion [71], we transformed the data using compositional data analyses with a natural logarithm [72]. We also substituted the zero values with small values (10^−6^) as suggested by Aebischer et al., 1993 [72]. We used Student’s t-test to compare groups. In the tests between shed skin vs. live individual samples we did not use the same individuals’ samples in both groups, to avoid repeated measures.

As there was no significant difference found between males and females’ skin lipids chemical composition with only three exceptions (see Section 2), we grouped samples from both sexes in the following analyses-skin vs. live individuals and live pre-reproductive phase individuals vs. live reproductive phase individuals. We did not include the shed skin samples in the statistical analyses between the pre-reproductive and reproductive periods, since the skins were shed at a different time of the year (see Section 4.1).

We also performed principal component analysis (PCA) to measure the effect of the variables on the principal components.

We tested for correlations between the number of detected compounds and the snout–vent length (SVL) and body mass of the live individuals, using the nonparametric Spearman’s R.

For all statistical procedures, we set the significance level at *p* ≤ 0.05.

## 5. Conclusions

The detected compounds of the skin secretion complex of *V. ammodytes* are similar to what we expected and what is described in the literature for other species. However, some major differences should be taken into consideration and studied further. The presence of only two main methyl ketones was undescribed before. The lack of a significant difference for all of the detected compounds between males and females should be investigated further. The results of the study suggest that chemical communication in *V. ammodytes* involves much less compounds compared to what has been described for other species.

## Figures and Tables

**Table 1:** Alkanes found in Vipera ammodytes samples (*n*). Values found in a single sample represent the relative percent of the compound; otherwise, we present the values as (median (minimum–maximum), number of samples in which the compound was present). Samples can belong to multiple groups. Statistically significant differences between relative percent of alkanes from shed skin and live specimens are denoted next to the compound formula as * for *p* < 0.05, **—*p* < 0.01, and ***—*p* < 0.001; differences between males and females are denoted, using ^, ^^, and ^^^; differences between the pre-reproductive and the reproductive phases are denoted using #, ##, and ###. Specific values for the tests are provided in Supplementary Material 3.

Compound	Skin (*n* = 10)	Live (*n* = 27)	Male (*n* = 13)	Female (*n* = 14)	Pre-Reproductive (*n* = 7)	Reproductive (*n* = 20)
*n*-Decane	–	1.78	1.78	–	–	1.78
*n*-Dodecane *** ^ #	0.96 (0.81–1.1), 2	4.25 (0.17–13.91), 20	3.34 (0.35–13.91), 12	5.29 (0.17–13.71), 8	6.67 (2.3–13.78), 7	2.18 (0.17–13.91), 13
*n*-Tridecane	–	0.31 (0.28–0.55), 5	0.38 (0.31–0.55), 3	0.29 (0.28–0.29), 2	0.38 (0.28–0.55), 3	0.3 (0.29–0.31), 2
*n*-Tetradecane ***	0.7 (0.01–2.93), 4	1.74 (0.03–19.39), 24	1.98 (0.16–19.39), 12	1.74 (0.03–18.44), 12	10.62 (1.54–19.39), 7	1.4 (0.03–18.44), 17
*n*-Pentadecane #	0.05 (0.03–0.06), 2	0.37 (0.11–0.67), 10	0.31 (0.11–0.42), 6	0.39 (0.34–0.67), 4	0.36 (0.11–0.4), 5	0.4 (0.18–0.67), 5
Cyclododecane #	0.06	0.18 (0.17–0.19), 2	0.17	0.19	0.18 (0.17–0.19), 2	–
*n*-Hexadecane ** ^ ##	1.33 (0.01–3), 3	1.08 (0.01–18.74), 20	3.24 (0.04–18.74), 12	0.98 (0.01–18.65), 8	15.77 (0.35–18.74), 7	0.48 (0.01–18.65), 13
*n*-Heptadecane	0.02 (0.01–0.04), 7	0.36 (0.02–2.53), 16	0.28 (0.02–0.65), 10	0.6 (0.19–2.53), 6	0.41 (0.22–0.65), 6	0.28 (0.02–2.53), 10
*n*-Octadecane * ^ ##	0.99 (0.03–2.28), 3	0.93 (0.04–16.5), 17	3.3 (0.04–16.04), 11	0.74 (0.13–16.5), 6	10.68 (0.15–12.71), 7	0.43 (0.04–16.5), 10
*n*-Nonadecane ##	0.03 (0.01–0.1), 5	0.33 (0.01–24.45), 16	0.28 (0.14–4.65), 9	0.43 (0.01–24.45), 7	0.35 (0.18–4.65), 7	0.25 (0.01–24.45), 9
Eicosane ##	0.07 (0.01–1.27), 6	1.26 (0.06–10.67), 18	1.51 (0.12–10.39), 11	1.09 (0.06–10.67), 7	5.23 (0.33–10.39), 7	0.56 (0.06–10.67), 11
*n*-Heneicosane **	0.09 (0.02–0.34), 7	0.43 (0.11–11.52), 25	0.46 (0.15–2.07), 13	0.39 (0.11–11.52), 12	0.66 (0.18–2.07), 7	0.39 (0.11–11.52), 18
Docosane ***	0.4 (0.03–0.84), 3	1.78 (0.25–5.4), 25	2 (0.25–5.17), 13	1.62 (0.39–5.4), 12	2.83 (1.18–3.44), 7	1.62 (0.25–5.4), 18
Tricosane ***	0.06 (0.01–0.5), 7	4.17 (0.08–6.14), 26	3.52 (0.2–5.45), 13	4.75 (0.08–6.14), 13	3.52 (0.08–5.14), 7	4.75 (0.11–6.14), 19
Tetracosane ***	0.21 (0.01–1.68), 6	6.48 (0.89–12.37), 26	6.53 (0.89–9.78), 13	6.43 (0.97–12.37), 13	3.85 (0.91–8.94), 7	7.04 (0.89–12.37), 19
Pentacosane	0.15 (0.02–1.66), 8	10.79 (0.28–17.42), 23	10.79 (1.38–13.85), 11	11.96 (0.28–17.42), 12	6.45 (1.38–12.94), 5	11.8 (0.28–17.42), 18
Hexacosane ***	0.12 (0.04–1.75), 5	11.3 (0.2–19.63), 26	9.92 (0.45–15.19), 13	12.55 (0.2–19.63), 13	5.38 (0.2–14.3), 7	13.36 (1.57–19.63), 19
Heptacosane	0.13 (0.04–1.39), 9	13.5 (0.34–18.43), 24	11.27 (0.61–16.03), 12	14.25 (0.34–18.43), 12	7.45 (1.75–13.61), 5	13.79 (0.34–18.43), 19
Octacosane ***	0.31 (0.08–0.65), 3	7.32 (0.2–14.49), 26	6.74 (0.46–13.91), 13	7.75 (0.2–14.49), 13	1.07 (0.2–10.43), 7	10.09 (0.54–14.49), 19
Nonacosane	0.14 (0.04–0.67), 9	7.63 (0.04–12.92), 22	7.12 (0.04–12.92), 12	8.86 (1.99–11.66), 10	4.11 (0.04–7.43), 5	8.79 (0.62–12.92), 17
Triacontane *	0.05 (0.04–0.06), 2	2.28 (0.18–8.13), 18	1.77 (0.18–8.13), 9	2.34 (0.87–4.5), 9	1.77 (0.18–3.83), 5	2.34 (0.28–8.13), 13
Hentriacontane	0.11 (0.01–0.34), 7	2.33 (0.08–11.38), 18	2.43 (0.15–11.38), 11	2.31 (0.08–3.71), 7	1.45 (0.15–3.76), 4	2.39 (0.08–11.38), 14
Phytane	–	0.04	0.04	–	–	0.04

**Table 2:** Sterol lipids and ketones found in Vipera ammodytes samples (*n*). Values found in a single sample represent the relative percent of the compound; otherwise, we present the relative percent as (median (minimum–maximum) number of samples in which the compound was present). Samples can belong to multiple groups (e.g., alive and male). Statistically significant differences between relative percent of sterol lipids and ketones from shed skin and live specimens are denoted next to the compound formula as * for *p* < 0.05, **—*p* < 0.01, and ***—*p* < 0.001; differences between males and females are denoted using ^, ^^, and ^^^; differences between the pre-reproductive and the reproductive phases are denoted similarly, using #, ##, and ###. Specific values for the tests are provided in Supplementary Material 3.

Compound	Skin (*n* = 10)	Live (*n* = 27)	Male (*n* = 13)	Female (*n* = 14)	Pre-Reproductive (*n* = 7)	Reproductive (*n* = 20)
2-Pentadecanone,6,10,14-trimethyl- (Hexahydrofarnesyl acetone; Phytone)	0.01	0.16		0.16	0.16	–
Hexadecanoic acid (Palmitic acid) ***	0.02	0.16 (0.09–0.23), 2	0.16 (0.09–0.23), 2	–	0.23	0.09
2-Pentacosanone *	0.13 (0.04–0.75), 7	0.58 (0.12–3.45), 6	0.22 (0.12–3.45), 5	0.94	0.79 (0.22–1.35), 2	0.56 (0.12–3.45), 4
2-Heptacosanone	2.43	0.31 (0.15–0.53), 4	0.31 (0.26–0.36), 2	0.34 (0.15–0.53), 2	–	0.31 (0.15–0.53), 4
Cholesterol myristate (Tetradecanoic acid, cholesteryl ester)	0.08 (0.04–0.11), 3	0.41 (0.15–0.94), 5	0.76 (0.15–0.94), 3	0.35 (0.28–0.41), 2	0.94	0.35 (0.15–0.76), 4
Cholesterol benzoate (Benzoic acid, cholesteryl ester)	0.45 (0.33–0.5), 3	0.8 (0.15–1.24), 4	0.87 (0.49–1.24), 2	0.63 (0.15–1.11), 2	1.24	0.49 (0.15–1.11), 3
Cholesta-4,6-dien-3-ol **	0.2 (0.09–0.4), 4	0.49	0.49	–	–	0.49
Cholesta-3,5-diene	0.22 (0.13–0.45), 7	0.51 (0.11–5.82), 12	0.31 (0.15–5.82), 7	0.69 (0.11–2.57), 5	3.03 (0.23–5.82), 2	0.51 (0.11–2.57), 10
Cholestan-3-one *	1.13 (1.08–1.18), 2	–	–	–	–	–
Desmosterol (Cholesta-5,24-dien-3-ol) *	0.72 (0.49–1.29), 4	0.56 (0.03–1.09), 2	–	0.56 (0.03–1.09), 2	–	0.56 (0.03–1.09), 2
Cholest-4-en-3-one	16.17	–	–	–	–	–
2-Tritriacontanone	–	1.37	1.37	–	–	1.37
Sitosterol	0.83	–	–	–	–	–
Cholesta-4,6-diene-3-one ***	0.71 (0.6–1.14), 5	–	–	–	–	–
Stigmast-4-en-3-one	–	0.56	–	0.56	–	0.56

**Table 3:** Aldehydes, fatty acids, fatty alcohols, esters and phenols found in Vipera ammodytes samples (*n*). Values found in a single sample represent the relative percent of the compound; otherwise, we present the relative percent as (median (minimum–maximum) number of samples in which the compound was present). Samples can belong to multiple groups (e.g., alive and male). Statistically significant differences between the relative percent of aldehydes, fatty acids, fatty alcohols, esters and phenols from shed skin and live specimens are denoted next to the compound formula as * for *p* < 0.05, **—*p* < 0.01, and ***—*p* < 0.001; differences between males and females are denoted using ^, ^^, and ^^^; differences between the pre-reproductive and the reproductive phases are denoted similarly, using #, ##, and ###. Specific values for the tests are provided in Supplementary Material 3.

Compound	Skin (*n* = 10)	Live (*n* = 27)	Male (*n* = 13)	Female (*n* = 14)	Pre-Reproductive (*n* = 7)	Reproductive (*n* = 20)
Decanal	–	0.17 (0.12–0.66), 4	0.15	0.19 (0.12–0.66), 3	0,12	0.19 (0.15–0.66), 3
Tridecanal	–	0.09	–	0.09	0.09	–
2-Nonen-1-ol	0.16	0.11	–	0.11	0.11	–
Phenol, ditert-butyl- #		0.05 (0.04–0.06), 2	0.06	0.04	0.05 (0.04–0.06), 2	–
Tetradecanol	0.02 (0.01–0.03), 2	0.41 (0.25–0.57), 2	0.25	0.57	–	0.41 (0.25–0.57), 2
Pentadecanol *	0.02 (0.01–0.03), 5	0.12 (0.06–0.22), 3	0.12 (0.06–0.22), 3	–	–	0.12 (0.06–0.22), 3
Methyl palmitate (Hexadecanoic acid, methyl ester-)	–	0.14	0.14	–	–	0.14
Benzoic acid, butyl ester-	–	–	–	–	–	–
Hexadecanoic acid (Palmitic acid) ***	0.21 (0.01–1.05), 8	0.22 (0.18–1.23), 3	0.22 (0.18–1.23), 3	–	0.71 (0.18–1.23), 2	0.22
Octadecanal (Stearaldehyde)	0.03 (0.01–0.05), 2	0.29 (0.12–0.45), 2	0.29 (0.12–0.45), 2	–	0.45	0.12
Octadecanoic acid (Stearic acid ***	0.43 (0.08–1.55), 5	–	–	–	–	–
9-Octadecenoic acid ***	1.14 (0.04–8.98), 6	–	–	–	–	–
9,12-Ocatdecadienoic acid ***	0.08 (0.01–4.72), 7	–	–	–	–	–
Docosanal **	0.03 (0.02–0.06), 4	0.53	0.53	–	0.53	–
Squalene	0.11 (0.03–0.26), 6	1.38 (0.21–70.48), 17	1.03 (0.28–12.8), 8	2 (0.21–70.48), 9	0.62 (0.31–3.24), 4	1.78 (0.21–70.48), 13
a-Tocopherol (Vitamin E) *	0.3 (0.09–0.51), 2	–	–	–	–	–
Tocopherol (isomer)	4.90	–	–	–	–	–
Docosanol, formate	0.09	–	–	–	–	–

## Supplementary Materials

The raw data with ecological and morphometric characteristics of the individuals and the detected compounds are available online as Supplementary Materials.
