# Supplementary material for: Analyses of Skin Secretions of Vipera ammodytes (Linnaeus, 1758) (Reptilia: Serpentes), with Focus on the Complex Compounds and Their Possible Role in the Chemical Communication
Source: Molecules. 2020 Aug 9;25(16):3622. doi: 10.3390/molecules25163622 (PMC7465031; doi:10.3390/molecules25163622)
Supplement: Supplementary file 1 [file molecules-25-03622-s001.zip › molecules-871751-Supplementary Materials/Supplementary Material_1.docx]

Analyses of Skin Secretions of *Vipera ammodytes* (Linnaeus, 1758) (Reptilia: Serpentes), with Focus on the Complex Compounds and Their Possible Role in the Chemical Communication

Kostadin Andonov ^1,^*, Angel Dyugmedzhiev ^1^, Simeon Lukanov ^1^, Miroslav Slavchev ^1^, Emiliya Vacheva ^2^, Nikola Stanchev ^3^, Georgi Popgeorgiev ^2,4^, Deyan Duhalov ^5^, Yurii V. Kornilev ^2,6^, Daniela Nedeltcheva-Antonova ^7^ and Borislav Naumov ^1^

^1^ Institute of Biodiversity and Ecosystem Research-BAS, Sofia, Bulgaria, 2 Gagarin Street, 1113 Sofia, Bulgaria; angel_diugmedjiev@abv.bg (A.D.); simeon_lukanov@abv.bg (S.L.); slmiro@abv.bg (M.S.); herpetology_bg@yahoo.com (B.N.)

^2^ National Museum of Natural History-BAS, Sofia, Bulgaria, 1 Tsar Osvoboditel Blvd., 1000 Sofia, Bulgaria; emilia.vacheva@gmail.com (E.V.); georgi.popgeorgiev@gmail.com (G.P.); yukornilev@gmail.com (Y.V.K.)

^3^ Sofia University “St. Kliment Ohridski”, Sofia, Bulgaria, 15 Tsar Osvoboditel Blvd., 1504 Sofia, Bulgaria; nickolastanchev@abv.bg

^4^ Bulgarian Society for the Protection of Birds/BirdLife Bulgaria, Yavorov Complex, Bl. 71, Vh. 4, PO Box 50, 1111 Sofia, Bulgaria

^5^ Independent researcher, Vrabnica 1 Complex, Bl. 538, Vh. V, ap. 47, 1229 Sofia, Bulgaria; deyan_duchalov@abv.bg

^6^ Integrative Zoology, Department of Evolutionary Biology, Faculty of Life Sciences, University of Vienna, Althanstrasse 14, 1090 Vienna, Austria

^7^ Institute of Organic Chemistry with Centre of Phytochemistry-BAS, Sofia, Bulgaria, 9 Acad. G. Bonchev Street, 1113 Sofia, Bulgaria; dantonova@orgchm.bas.bg

***** Correspondence: [k_andonov91@abv.bg](mailto:k_andonov91@abv.bg)

**Table 1.** Detected compounds in the skin secretion extracts of V. ammodytes specimens via coupled GC-MS.

| **Compound Detected** | **t_R_, min** | **Identification** | **Molecular Formula** | **Type** |
| --- | --- | --- | --- | --- |
| *n*-Decane | 6,18 | Reference compound | C_10_H_22_ | Straight-chain alkane |
| *n*-Dodecane | 8,14 | Reference compound | C_12_H_26_ | Straight-chain alkane |
| *n*-Tridecane | 9,89 | Reference compound | C_13_H_28_ | Straight-chain alkane |
| Decanal | 10,20 | NIST14, Wiley07 | C_10_H_20_O | Aldehyde |
| *n*-Tetradecane | 11,43 | Reference compound | C_14_H_30_ | Straight-chain alkane |
| *n*-Pentadecane | 12,79 | Reference compound | C_15_H_32_ | Straight-chain alkane |
| Tridecanal | 13,31 | NIST14, Wiley07 | C_13_H_26_O | Aldehyde |
| Cyclododecane | 13,98 | NIST14, Wiley07 | C_12_H_24_ | Cycloalkane |
| *n*-Hexadecane | 14,18 | Reference compound | C_16_H_34_ | Straight-chain alkane |
| 5,9-Undecadien-2-one, 6,10-dimethyl- | 14,50 | NIST14, Wiley07 | C_13_H_22_O | Monoterpene ketone |
| 2-Nonen-1-ol | 14,57 | NIST14, Wiley07 | C_9_H_18_O | Fatty alcohol |
| *n*-Heptadecane | 15,64 | Reference compound | C_17_H_36_ | Straight-chain alkane |
| Phenol, ditert-butyl- | 15,75 | NIST14, Wiley07 | C_14_H_22_O | Phenol |
| Tetradecanol | 16,09 | NIST14, Wiley07 | C_14_H_30_O | Fatty alcohol |
| *n*-Octadecane | 17,18 | Reference compound | C_18_H_38_ | Straight-chain alkane |
| *n*-Nonadecane | 18,77 | Reference compound | C_19_H_40_ | Straight-chain alkane |
| Pentadecanol | 19,34 | NIST14, Wiley07 | C_15_H_32_O | Fatty alcohol |
| 2-Pentadecanone, 6,10, 14-trimethyl- (Hexahydrofarnesyl acetone; Phytone) | 19,51 | NIST14, Wiley07 | C_18_H_36_O | Methyl ketone, Isoprenoid |
| Eicosane | 20,42 | Reference compound | C_20_H_42_ | Straight-chain alkane |
| Methyl palmitate (Hexadecanoic acid, methyl ester-) | 21,08 | NIST14, Wiley07 | C_17_H_34_O_2_ | Fatty acid methyl ester |
| Benzoic acid, butyl ester- | 21,18 | NIST14, Wiley07 | C_11_H_14_O_2_ | Benzoic acid ester |
| *n*-Heneicosane | 22,05 | Reference compound | C_21_H_44_ | Straight-chain alkane |
| Hexadecanoic acid (Palmitic acid) | 22,15 | NIST14, Wiley07 | C_16_H_32_O_2_ | Fatty acid |
| Docosane | 23,69 | Reference compound | C_22_H_46_ | Straight-chain alkane |
| Octadecanal (Stearaldehyde) | 24,37 | NIST14, Wiley07 | C_18_H_36_O | Fatty aldehyde |
| Tricosane | 25,28 | Reference compound | C_23_H_48_ | Straight-chain alkane |
| Octadecanoic acid (Stearic acid) | 25,56 | NIST14, Wiley07 | C_18_H_36_O_2_ | Fatty acid |
| 9-Octadecenoic acid | 25,64 | NIST14, Wiley07 | C_18_H_34_O_2_ | Fatty acid |
| 9,12-Ocatdecadienoic acid | 25,92 | NIST14, Wiley07 | C_18_H_32_O_2_ | Fatty acid |
| Tetracosane | 26,86 | Reference compound | C_24_H_50_ | Straight-chain alkane |
| Docosanal | 27,58 | NIST14, Wiley07 | C_22_H_44_O | Fatty aldehyde |
| Pentacosane | 28,38 | Reference compound | C_25_H_52_ | Straight-chain alkane |
| 4,8,12-Trimethyltridecan-4-olide ([5-(4,8-Dimethylnonyl)-5-methyldihydro-2(3H)-furanone](https://pubchem.ncbi.nlm.nih.gov/substance/163241086)) | 29,46 | NIST14, Wiley07 | C_16_H_30_O_2_ | Ketone with furane ring |
| Hexacosane | 29,85 | Reference compound | C_26_H_54_ | Straight-chain alkane |
| Heptacosane | 31,27 | Reference compound | C_27_H_56_ | Straight-chain alkane |
| Octacosane | 32,66 | Reference compound | C_28_H_58_ | Straight-chain alkane |
| 2-Pentacosanone | 33,28 | NIST14, Wiley07 | C_25_H_50_O | Methyl ketone |
| Nonacosane | 34,12 | Reference compound | C_29_H_60_ | Straight-chain alkane |
| Triacontane | 35,63 | Reference compound | C_30_H_62_ | Straight-chain alkane |
| Squalene | 35,76 | NIST14, Wiley07 | C_30_H_50_ | Triterpene |
| 2-Heptacosanone | 36,45 | NIST14, Wiley07 | C_27_H_54_O | Methyl ketone |
| Hentriacontane | 37,27 | Reference compound | C_31_H_64_ | Straight-chain alkane |
| Cholesterol myristate (Tetradecanoic acid, cholesteryl ester) | 37,42 | NIST14, Wiley07 | C_41_H_72_O_2_ | Sterol lipid |
| Cholesterol benzoate (Benzoic acid, cholesteryl ester) | 37,89 | NIST14, Wiley07 | C_34_H_50_O_2_ | Sterol lipid |
| Cholesta-4,6-dien-3-ol | 37,91 | NIST14, Wiley07 | C_27_H_44_O | Sterol lipid |
| Cholesta-3,5-diene | 38,11 | NIST14, Wiley07 | C_27_H_44_ | Sterol lipid |
| Phytane | 39,04 | NIST14, Wiley07 | C_20_H_42_ | Isoprenoid alkane |
| a-Tocopherol (Vitamin E) | 39,43 | NIST14, Wiley07 | C_29_H_50_O_2_ | Methylated phenol with chromane  (benzo dihydropyran) ring |
| Tocopherol (isomer) | 44,05 | NIST14, Wiley07 | C_29_H_50_O_2_ | Methylated phenol with chromane  (benzo dihydropyran) ring |
| Cholesterol | 44,05 | NIST14, Wiley07 | C_27_H_46_O | Sterol lipid |
| 2-Hentriacontanone | 44,05 | NIST14, Wiley07 | C_31_H_62_O | Methyl ketone |
| Cholestan-3-one | 44,86 | NIST14, Wiley07 | C_27_H_46_O | Sterol lipid |
| Desmosterol (Cholesta-5,24-dien-3-ol) | 45,63 | NIST14, Wiley07 | C_27_H_44_O | Sterol lipid |
| Cholest-4-en-3-one | 46,52 | NIST14, Wiley07 | C_27_H_44_O | Sterol lipid |
| 2-Tritriacontanone | 46,52 | NIST14, Wiley07 | C_33_H_66_O | Methyl ketone |
| Sitosterol | 46,85 | NIST14, Wiley07 | C_29_H_50_O | Sterol lipid |
| Cholesta-4,6-diene-3-one | 47,07 | NIST14, Wiley07 | C_27_H_42_O | Sterol lipid |
| Stigmast-4-en-3-one | 48,72 | NIST14, Wiley07 | C_29_H_48_O | Sterol lipid |
| Docosanol, formate | 52,88 | NIST14, Wiley07 | C_23_H_46_O_2_ | Ester |
